# A Comparative Study of the Innate Humoral Immune Response to Avian Influenza Virus in Wild and Domestic Mallards

**DOI:** 10.3389/fmicb.2020.608274

**Published:** 2020-11-30

**Authors:** Jacintha G. B. van Dijk, Josanne H. Verhagen, Arne Hegemann, Conny Tolf, Jenny Olofsson, Josef D. Järhult, Jonas Waldenström

**Affiliations:** ^1^Centre for Ecology and Evolution in Microbial Model Systems, Linnaeus University, Kalmar, Sweden; ^2^Department of Biology, Lund University, Ecology Building, Lund, Sweden; ^3^Zoonosis Science Center, Department of Medical Sciences, Uppsala University, Uppsala, Sweden

**Keywords:** Anas platyrhynchos, baseline immune function, complement, haptoglobin, infectious disease, innate humoral immunity, natural antibody, sentinel

## Abstract

Domestic mallards (*Anas platyrhynchos domesticus*) are traditionally used as a model to investigate infection dynamics and immune responses to low pathogenic avian influenza viruses (LPAIVs) in free-living mallards. However, it is unclear whether the immune response of domestic birds reflects the response of their free-living counterparts naturally exposed to these viruses. We investigated the extent to which the innate humoral immune response was similar among (i) wild-type domestic mallards in primary and secondary infection with LPAIV H4N6 in a laboratory setting (laboratory mallards), (ii) wild-type domestic mallards naturally exposed to LPAIVs in a semi-natural setting (sentinel mallards), and (iii) free-living mallards naturally exposed to LPAIVs. We quantified innate humoral immune function by measuring non-specific natural antibodies (agglutination), complement activity (lysis), and the acute phase protein haptoglobin. We demonstrate that complement activity in the first 3 days after LPAIV exposure was higher in primary-exposed laboratory mallards than in sentinel and free-living mallards. LPAIV H4N6 likely activated the complement system and the acute phase response in primary-exposed laboratory mallards, as lysis was higher and haptoglobin lower at day 3 and 7 post-exposure compared to baseline immune function measured prior to exposure. There were no differences observed in natural antibody and haptoglobin concentrations among laboratory, sentinel, and free-living mallards in the first 3 days after LPAIV exposure. Our study demonstrates that, based on the three innate humoral immune parameters measured, domestic mallards seem an appropriate model to investigate innate immunology of their free-living counterparts, albeit the innate immune response of secondary-LPAIV exposed mallards is a better proxy for the innate immune response in pre-exposed free-living mallards than that of immunologically naïve mallards.

## Introduction

Low pathogenic avian influenza virus (LPAIV) is a zoonotic pathogen that circulates naturally in wild birds (Webster et al., 1992; Hurt et al., 2017). Ducks are considered a main LPAIV reservoir, which are frequently infected with these virus and harbor the majority of subtypes as detected in birds to date (H1-H16, N1-N9) (Olsen et al., 2006; Olson et al., 2014). Spill-over of LPAIV subtypes H5 and H7 from wild birds to poultry can result in massive outbreaks, sometimes involving the culling of millions of domestic birds, when LPAIVs evolve into highly pathogenic avian influenza viruses (Alexander, 2007). Mallards (*Anas platyrhynchos*) are suggested to play an important role in the transmission of LPAIVs to domestic birds (chickens, turkeys, and ducks) and their wild counterparts, partly due to their great abundance (~19 million individuals), worldwide distribution, and preference for human-influenced environments (Wille et al., 2017). LPAIV infections in mallards cause no apparent tissue damage or disease signs (Kuiken, 2013). Furthermore, it has been suggested that LPAIVs do not induce strong innate immune responses in this reservoir host (Magor, 2011; Evseev and Magor, 2019). The influenza virus sensor *RIG-I* plays a key role in the innate immune response of domestic mallards (*A. p. domesticus*) by clearing LPAIV infection (Barber et al., 2010), albeit *RIG-I* expression, as well as the resistance gene *Mx* and several other innate immune genes are transient and only weakly upregulated in response to LPAIV exposure in domestic mallards (Vanderven et al., 2012; Helin et al., 2018; Fleming-Canepa et al., 2019). LPAIV infection does not induce pro-inflammatory cytokines in tissues and cells of domestic mallards (Evseev and Magor, 2019). Although domestic mallards are traditionally used as a model to investigate the innate immune response to LPAIV in free-living mallards, it remains unclear how well these results reflect the immune response of their free-living counterparts upon natural exposure.

Most of our knowledge on the innate immune response of mallards comes from experimental LPAIV infection studies in the laboratory using Pekin ducks or wild-type domestic mallards. The Pekin duck is the most widely used commercial duck breed for meat and eggs (Cherry and Morris, 2008), and wild-type mallards are bred on farms for slaughter and/or to be released into the wild for hunting (Champagnon et al., 2009). There are substantial differences between Pekin ducks and free-living mallards in absolute rate of bone growth, body mass and composition, and flight feathers (Cherry and Morris, 2008). There is clear genetic differentiation between wild-type domestic mallards and free-living mallards, as well as phenotypic differences (Söderquist et al., 2014, 2017). Domestication usually leads to changes in the phenotype of an animal, both because selection on specific desired traits and due to changes in space, food, predation, social environment, and genotype due to inbreeding and genetic drift (Price, 1999). Selection can also induce changes to the immune system (Lamont et al., 2003); therefore, Pekin ducks and wild-type domestic mallards may also differ in their innate immune response to pathogens, such as LPAIVs, compared to free-living mallards. Besides genetic makeup, environmental conditions may also affect immune function (Gomez et al., 2005; Calisi and Bentley, 2009; Hegemann et al., 2012). Pekin ducks and wild-type mallards used in experimental LPAIV infection studies are typically held in enclosed, constant and relatively clean environments in the lab, where they have access to unlimited food and are kept free from other pathogens than LPAIV. Their free-living counterparts on the other hand, are free to move, exposed to variable environmental conditions (including weather fluctuations, food shortages, and predation) and continuously exposed to a variety of parasites. Therefore, it remains unclear how well the innate immune response of Pekin ducks or wild-type mallards upon experimental LPAIV exposure in captivity reflects the innate immune response of free-living mallards upon natural LPAIV exposure. Knowing whether the innate immune response of domestic mallards upon LPAIV exposure is a good proxy for the immune response in free-living mallards is of critical importance if one would like to incorporate host immunity in epidemiological models. Failure to include accurate measures of immunity into these models can result in poor estimates of transmission rates and epidemic probabilities in wild bird populations.

Hence, the aim of this study was to investigate the extent to which the innate immune response upon LPAIV exposure of wild-type domestic mallards is comparable to that of free-living mallards. We compared the innate humoral immune response between (i) wild-type domestic mallards experimentally exposed once (primary-exposed) or twice (secondary-exposed) to LPAIVs in a laboratory setting (hereafter called laboratory mallards), (ii) wild-type domestic mallards naturally exposed to LPAIVs in a semi-natural setting (hereafter called sentinel mallards), and (iii) free-living mallards naturally exposed to LPAIV in a natural setting. This study design enabled us to explore whether differences in innate immune response were associated with domestication (laboratory/sentinel vs. free-living) or with environmental conditions and infection history (laboratory vs. sentinel/free-living). We hypothesized that (immunologically naïve) laboratory mallards would show a stronger innate immune response upon LPAIV exposure compared to sentinel and free-living mallards, who have been pre-exposed to various LPAIVs and other pathogens in nature (Wille et al., 2013, 2015). We quantified innate humoral immune function by measuring nonspecific natural antibodies (agglutination), natural antibody-mediated complement activation (lysis), and the acute phase protein haptoglobin (or a functional equivalent, see Matson et al., 2012) in mallard serum. Red blood cell agglutination and complement-mediated lysis reflect responses to antigens (viruses, bacteria, and toxins) and are driven by natural antibodies and the complement system, respectively (Matson et al., 2005; Uribe et al., 2011). Lysis reflects the interaction of complement and natural antibodies, while agglutination results from natural antibodies alone (Matson et al., 2005). Natural antibodies are produced in the absence of exogenous antigenic stimulation and are likely unaffected by prior infection (Ochsenbein and Zinkernagel, 2000), whereas complement, a group of proteins involved in inflammation, can be activated directly by pathogens or indirectly by antigen-bound antibodies, such as immunoglobulin (IgM; via the classical complement pathway; Müller-Eberhard, 1988). Haptoglobin is a protein of the acute phase response that binds free hemoglobin to prevent it from providing nutrients to pathogens. The release of haptoglobin is regulated by the liver, and often activated by cytokines of the interleukin 1 family during the onset of infection (Murata et al., 2004; Quaye, 2008). Normally this protein circulates at low concentrations in blood, but it has been shown to increase rapidly in response to infection, inflammation, or trauma in avian species (Delers et al., 1988; Millet et al., 2007; van de Crommenacker et al., 2010). These innate humoral immune parameters have been shown to play a role in activating the innate immune system in animals experimentally exposed to LPAIV. Natural antibodies (predominantly IgM) and complement work in concert to neutralize LPAIV H1N1 in laboratory mice (*Mus musculus domesticus*; Jayasekera et al., 2007; O’Brien et al., 2011). Haptoglobin is mounted in response to LPAIV H5N2, H5N3, and H9N2 infection in chickens (*Gallus domesticus*; Sylte and Suarez, 2012; Dadras et al., 2014). These innate immune parameters can be assessed in live birds, requiring only a single small blood sample used for multiple assays, and therefore ideal for comparative immunological studies (Matson et al., 2005, 2012; Hegemann et al., 2017). We assessed baseline immune function for each innate immune parameter prior to LPAIV exposure in laboratory and sentinel mallards to compare baselines among the different groups and to determine the magnitude of the innate immune response.

## Materials and Methods

### Experimental Infection Study

Wild-type domestic mallards (3–5 months of age, male) were housed in HEPA filtered rooms (14 m^2^) with negative air pressure at a BSL2 animal facility at the Swedish National Veterinary Institute, with a 12 h day–night cycle. Birds had access to a pool for swimming, and feed and drinking water *ad libitum*.

**Figure 1:**
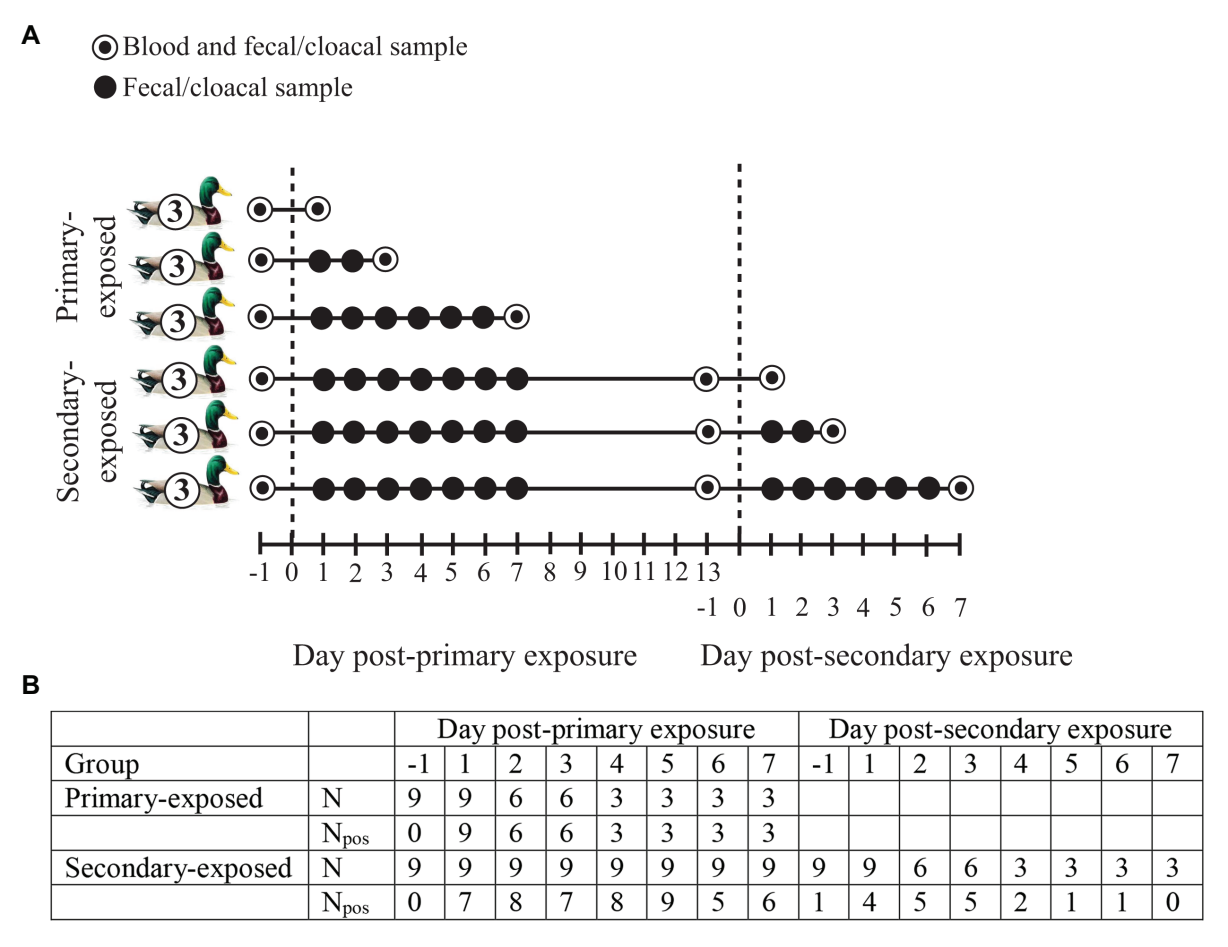
**(A)** Experimental design of wild-type domestic mallards exposed once (primary-exposed group) or twice (secondary-exposed group) via contact with low pathogenic avian influenza virus (LPAIV) H4N6-inoculated mallards in a laboratory setting. The dashed line represents the day of virus exposure. **(B)** For each post-exposure day, the number of fecal or cloacal samples collected (*N*) is depicted, together with the number of samples that tested positive for influenza A viruses based on viral RNA detection (N_pos_).

In October 2017, a total of 24 wild-type domestic mallards were randomly distributed into two groups: (i) inoculated mallards (*N* = 6) and (ii) contact mallards (*N* = 18). The latter category consisted of two groups of nine birds each, denoted primary-exposed and secondary-exposed groups (Figure 1A). The inoculated group of mallards were inoculated directly into the esophagus with 1 ml of in total 10^6^ EID_50_ of A/Mallard/Sweden/80148/2009 (H4N6) LPAIV, either 2 days prior to primary exposure of contact mallards (*N* = 3), or 2 days prior to secondary exposure of contact mallards (*N* = 3). A LPAIV H4N6 was selected, as H4 is a very common subtype and H4N6 is a very common subtype combination in mallards, frequently isolated from free-living mallards at Ottenby in Sweden, the study location of our natural infection study (Latorre-Margalef et al., 2014). The inoculated mallards were used to infect the contact mallards from day 3 after inoculation onward by sharing the experimental room for 4 days. The primary- and secondary-exposed group of contact mallards (*N* = 18) were exposed to three inoculated mallards at day 0 post-primary exposure. Only the secondary-exposed group (*N* = 9) were exposed for a second time to three inoculated mallards at day 0 post-secondary exposure. The data used in this study is based on the contact mallards (Figure 1A). Data of the inoculated mallards has not been included due to different exposure routes.

Fecal or cloacal samples for detection of avian influenza virus (AIV) were collected from the primary-exposed and secondary-exposed group of contact mallards at day 1 (i.e., 1 day before exposure), and day 1–7 post-primary and secondary exposure (Figure 1A). A blood sample from the brachial vein was collected from the primary-exposed and secondary-exposed group at day 1 post-primary exposure to assess primary-baseline immune function. Another blood sample was collected from the secondary-exposed group at day 1 post-secondary exposure to determine whether the innate immune response after primary exposure had returned to baseline levels prior to secondary exposure. To investigate the immune response upon LPAIV exposure, a blood sample from the jugular vein was collected from three individuals per time point: at day 1, 3, and 7 post-primary exposure (primary-exposed group) and at day 1, 3, and 7 post-secondary exposure (secondary-exposed group). Blood was collected immediately after birds were euthanized by exsanguination under isoflurane anesthesia. Prior to the start of the experiment, all individuals in the primary-exposed and secondary-exposed group tested negative for AIV RNA and AIV-specific antibodies.

### Natural Infection Study

From mid-September until mid-December 2017, free-living migratory mallards (all ages, male, and female) were caught daily in a stationary funnel live-trap at Ottenby in southeast Sweden (56°12'N, 16°24'E), a major stopover site for migratory waterfowl along the Northwest European flyway (Scott and Rose, 1996). In the northern hemisphere, LPAIV infection in mallard populations is highest in autumn (Latorre-Margalef et al., 2014; van Dijk et al., 2014). The trap was located partly in water and partly on land in a brackish lagoon. Free-living mallards were attracted by grain placed at the entrances and inside the trap. The trap was emptied daily and captured birds marked with a metal ring, sexed, aged (juvenile: <1 year, adult: >1 year) and sampled for AIV detection. For virus detection, cloacal samples or freshly deposited fecal samples in single-use cardboard boxes were collected using sterile cotton swabs, placed individually in viral transport media and stored at −80°C within 1–5 h of collection. In addition, once a week on the same day, blood samples (<1 ml, <2% of the circulating blood volume) were collected from the brachial vein of captured birds. For recaptured birds, we ensured that the blood samplings were at least 2 weeks apart for each individual. Blood was allowed to clot for approximately 6 h before centrifugation to separate serum and cell fractions (Hoye, 2012). Serum samples were stored at −20°C until analysis.

During the period that free-living mallards were captured, nine wild-type domestic mallards (~1.5 year, female) were housed in a separate compartment of the trap and sampled for AIV infection. Nylon mesh separated the sentinels from the free-living mallards, enabling not only water and natural food items (e.g., seeds, invertebrates) to move freely between their compartment and the surrounding environment, but also LPAIVs to be transmitted via water, splashes and droplets from free-living mallards. Half of the sentinel compartment consisted of water for drinking and swimming. Daily grain was provided and the birds had access to a small house for shelter. The birds were marked with a metal and a color ring, and their primary feathers were clipped to prevent the birds from flying. The sentinels had been in the trap since April 2017, but no samples for AIV detection had been collected before the start of the study. During the study period (85 days), we collected almost daily cloacal or freshly deposited fecal samples from the sentinels (seven birds: 79 samples; two birds: 78 samples, missing samples due to logistical reasons). On the same day free-living mallards were bled, we also collected blood samples from sentinels. To ensure that the blood samplings were at least 2 weeks apart for each individual, the sentinels were divided into two groups: five birds that were sampled in even weeks (*N* = 7 samples), and four birds sampled in odd weeks (*N* = 6 samples). Collection and storage of samples was analogous to that of free-living mallards. Prior to the study, all sentinels tested positive for AIV-specific antibodies.

### Virus Detection

All samples were analyzed at Linnaeus University, Kalmar, as described previously (Wille et al., 2014). In short, RNA from cloacal and fecal samples was extracted with a MagNA Pure 96 Extraction robot and the viral NA large volume kit (Roche Diagnostics GmbH, Mannheim, Germany). Influenza A viruses were detected using real-time reverse transcriptase PCR (RRT-PCR) assays targeting the matrix gene of the influenza A virus (Wille et al., 2014). Samples were considered positive for influenza A viruses if the cycle threshold values were <40.

### Immune Assays

Natural antibodies (measured as agglutination) and complement activity (measured as lysis) were quantified using a hemolysis–hemagglutination assay following Matson et al. (2005). This assay was developed to assess constitutive innate humoral immunity requiring a single blood (serum) sample. The natural antibodies measured by this assay are primarily pentameric IgM, because agglutination disappears when treating plasma with 2-mercaptoethanol, which should break the sulfur bonds that make monomeric IgY into polymeric IgM. All, or almost all, of the lysis measured by this assay with unheated serum is due to complement. Lysis is always lower than agglutination, indicating that immunoglobulin was not limiting for measurement of complement levels. We followed the assays as described in Matson et al. (2005), in which no 2-mercaptoethanol and/or heating controls were used. In brief, serum samples were serially diluted in microtitre plates and incubated with a 1% rabbit red blood cell suspension (Envigo Laboratories, Bicester, United Kingdom). Following incubation, plate images were recorded after 20 min for agglutination and after 90 min for lysis. Images of individual samples were randomized and scored at least twice to reduce errors, always blindly with respect to sample ID and experimental treatment. Agglutination and lysis were recorded as titers (−log^2^ of the last serum dilution for each reaction).

We quantified haptoglobin concentrations (mg ml^−1^) using a commercially available colorimetric assay kit (TP801; Tri-Delta Diagnostics, Boonton, NJ, United States). This functional assay quantifies the heme-binding capacity of sera. We followed the “manual method” instructions provided by the kit manufacturer with a few minor modifications following Matson et al. (2012). We measured absorbance at two wavelengths (450 and 630 nm) using a microplate reader (FLUOstar Omega, BMG Labtech), prior to the addition of the final reagent that initiated the color-change reaction. We used the pre-scan at the normal assay wavelength of 630 nm to correct for differences in serum color and cloudiness by subtracting pre-scan absorbance values from final absorbance values. Further, the 450 nm pre-scan was included as a covariate in the statistical analysis to correct for differences in sera sample redness, an indicator of hemolysis, which can affect the assay (Matson et al., 2012). The final absorbance values were measured at 630 nm.

The samples from laboratory mallards were analyzed in different batches than the sentinel and free-living mallard samples. However, the standard or positive control on each plate did not differ between the batches of the laboratory mallards vs. the sentinel and free-living mallards for agglutination [linear model (LM): *F*_1,107_ = 1.86, *p* = 0.176], lysis (*F*_1,107_ = 0.02, *p* = 0.885), and haptoglobin (*F*_1,5_ = 4.24, *p* = 0.095). Samples were randomized before lab work, and the work was done blindly with respect to sample ID.

All serum samples were checked for AIV-specific antibodies using a competitive ELISA designed to detect anti-nucleoprotein antibodies (Influenza A Ab Test; IDEXX Laboratories Europe, Hoofddorp, the Netherlands), following the manufacturer’s instructions. The absorbance was measured at 620 nm using a microplate reader (FLUOstar Omega, BMG Labtech). Signal-to-noise ratios (i.e., the absorbance of the samples divided by the mean absorbance of the negative control) <0.5 were considered positive for the presence of antibodies specific to AIV. All serum samples were analyzed at Linnaeus University.

### Data Analysis

All birds in this study were categorized as either LPAIV infected (i.e., viral RNA detected by PCR) or uninfected (i.e., no viral RNA detected by PCR) on the day of sampling. Agglutination, lysis, and haptoglobin were tested for collinearity using Pearson correlation (r). Only agglutination and lysis were significantly correlated, but r^2^ was weak (0.05) and therefore both variables were included as separate response variables in the analyses. Haptoglobin was LN-transformed to meet the assumption of normality.

We were able to link the immune parameters in laboratory, sentinel and free-living mallards to day-post LPAIV exposure. In laboratory mallards, it was known how many days after LPAIV exposure a blood sample was collected, i.e., day 1, 3, or 7 post-exposure, and therefore the immune parameters could simply be linked to day post-exposure. In sentinel and free-living mallards, it was more complicated to assess which post-exposure day the day of blood sampling was. It was essential that birds were resampled for LPAIVs daily between the day of infection and the day of blood sampling to ensure birds were not infected a second time in this time lag. Furthermore, to ensure that the day of LPAIV infection was indeed day 1 post-exposure, it was essential that birds were uninfected prior to the day of LPAIV infection. For example, on October 11, a blood sample was collected from a sentinel mallard, and based on the fecal or cloacal samples we had collected on October 8, 9, 10, and 11, we knew that the bird was uninfected on October 8 and got its infection on October 9. Hence, we knew that the day of blood sampling corresponded to day 3 post-LPAIV exposure, and therefore the natural antibodies, complement activity, and haptoglobin reflected the immune response 3 days after exposure.

For each of the four mallard groups, we conducted separate analyses for natural antibodies (agglutination), complement activity (lysis), and the acute phase protein haptoglobin. These innate humoral immune parameters were included as response variables in these separate models. For the primary-exposed and secondary-exposed laboratory mallards, we used a linear mixed model (LMM) to assess whether agglutination, lysis, and haptoglobin on day 1, 3, and 7 post-exposure differed from primary- or secondary-baseline, and differed among each other. The models included the immune parameter as response variable, day post-exposure (i.e., baseline, 1, 3, and 7 post-exposure) as explanatory variable (factor) and individual bird ID as random factor (i.e., random intercept with fixed mean; Bates et al., 2015).

For seven of the nine sentinel birds, we were able to assess whether agglutination, lysis, and haptoglobin at days 1–2, 3–4, and 6–8 post-exposure differed from baseline immune function and differed among each other. We lumped the following post-exposure days to increase sample size: day 1 (*N* = 2) and day 2 (*N* = 2); day 3 (*N* = 2) and day 4 (*N* = 3); day 6 (*N* = 1), day 7 (*N* = 1) and day 8 (*N* = 4). We assessed baseline immune function for agglutination, lysis, and haptoglobin by using sera collected when individuals tested negative for virus detection for at least 20 days (29 ± 3 days, *N* = 15), a period long enough for the innate immune response to return to values prior to exposure (Higgins et al., 1987; Lee et al., 2012). Baseline immune function per bird was based on one serum sample or on the average of several samples of the same bird. We performed a LMM for each immune parameter with day post-exposure (i.e., baseline, 1–2, 3–4, and 6–8; factor) as explanatory variable, month (factor) as covariate, and individual bird ID as random factor. Month was included in the models, because innate immune function may vary over the year (Hegemann et al., 2012). One individual had a sampling gap of a single day, but since this individual was LPAIV-negative 75 out of 78 days (96%), we assigned this bird as uninfected on this day.

During the study period, we collected samples from free-living mallards captured once (*N* = 8) or multiple times (*N* = 52), consisting of 15 females and 45 males (42 juveniles and 18 adults). The average (±SE) recapture rate was 16.5 (±2.3). LPAIV infection prevalence during the study period was calculated by using a sampling interval of at least 30 days for LPAIV-infected recaptured birds (van Dijk et al., 2014). We could link the innate immune response to day-post LPAIV exposure for 23 (three females, 20 males; 17 juveniles, six adult; *N* = 26) of 60 individuals: day 1 (*N* = 11), day 2 (*N* = 12), day 3 (*N* = 2), and day 4 (*N* = 1). We used LMs to assess whether agglutination, lysis, and haptoglobin varied between days 1, 2, and 3–4 post-exposure, with days 3 and 4 lumped to increase sample size. The model included the immune parameter as response variable, day-post exposure (i.e., day 1, 2, 3–4; factor) as explanatory variable, and month (factor) and antibody status (i.e., AIV-specific antibody-positive or -negative; factor) as covariates. AIV-specific antibody status was included as a covariate in the models to correct for differences in acquired humoral immunity to LPAIV between individuals (Evseev and Magor, 2019).

To compare the innate humoral immune response upon LPAIV exposure among primary-exposed laboratory, secondary-exposed laboratory, sentinel and free-living mallards, we combined the measurements of agglutination, lysis, and haptoglobin of day 1, 2, and 3 post-exposure for each group. Combining the first 3 days after exposure allowed for a good comparison between the groups, as the innate immune response early in the LPAIV infection (days 1–3) was measured most accurately for each group. We conducted LMMs that included the immune parameter as response variable, bird group (i.e., primary-exposed: *N* = 6, secondary-exposed: *N* = 5, sentinel: *N* = 6, free-living: *N* = 24) as explanatory variable, day-post exposure (factor) and antibody status (factor) as covariates and individual bird ID as random factor. To exclude the fact that differences between primary-exposed laboratory, secondary-exposed laboratory, and sentinel mallards were caused by differences in baseline immune function, we performed a LM to assess whether baseline values of agglutination, lysis, and haptoglobin varied between the three mallard groups.

Tukey’s *post hoc* tests were performed to detect differences in immune parameters between baseline immune function and days post-exposure, among days post-exposure and among primary-exposed laboratory, secondary-exposed laboratory, sentinel, and free-living mallards. All analyses were conducted using R 3.6.1 (R Development Core Team, 2014). Package lme4 was used to fit LMMs (Bates et al., 2015) and package multcomp was used to perform a Tukey’s *post hoc* test (Hothorn et al., 2008).

## Results

### Experimental Infection Study

**Figure 2:**
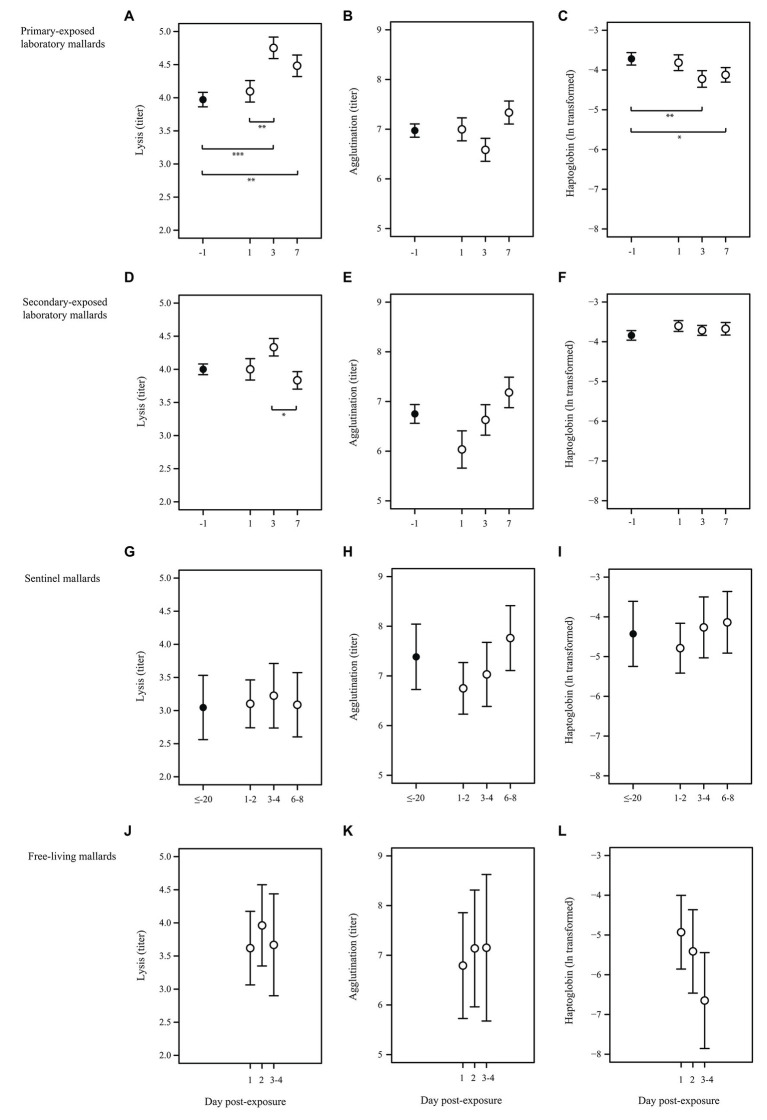
Characteristics of the innate humoral immune response upon LPAIV exposure in wild-type domestic mallards in a laboratory setting (laboratory mallards), wild-type domestic mallards in a semi-natural setting (sentinel mallards), and free-living mallards in a natural setting. Baseline immune function (black dots) and immune response upon LPAIV exposure (white dots) of lysis, agglutination, and haptoglobin (mean ± SE) in **(A–C)** primary-exposed laboratory mallards at day -1 (primary-baseline; *N* = 9), day 1 (*N* = 3), day 3 (*N* = 3), and day 7 (*N* = 3) post-H4N6 LPAIV exposure; **(D–F)** secondary-exposed laboratory mallards at day -1 (secondary-baseline; *N* = 8), day 1 (*N* = 2), day 3 (*N* = 3), and day 7 (*N* = 3) post-H4N6 LPAIV exposure; **(G–I)** sentinel mallards at day ≤–20 (baseline was based on a LPAIV-free period of at least 20 days; *N* = 7), day 1 and 2 (*N* = 4), day 3 and 4 (*N* = 5) and day 6, 7, and 8 (*N* = 6) post-natural LPAIV exposure; **(J–L)** free-living mallards at day 1 (*N* = 11), day 2 (*N* = 12), days 3 and 4 (*N* = 3) post-natural LPAIV exposure. Significant codes represent: ^***^ = *p* < 0.001; ^**^ = *p* < 0.01; ^*^ = *p* < 0.05.

All primary-exposed laboratory mallards (*N* = 9) were excreting LPAIV H4N6 daily from day 1 to day 7 post-exposure (Figure 1B). We collected blood samples from three individuals per time point, i.e., day 1, 3, and 7 post-primary exposure. Birds were negative for AIV-specific antibodies at day 1 (0/3) and positive for AIV-specific antibodies at day 3 (3/3) and day 7 (3/3) post-primary exposure. Complement activity (lysis) and the acute phase protein haptoglobin differed between primary-baseline (i.e., day -1 post-primary exposure) and the post-exposure days (*X*^2^ = 14.05, *p* = 0.003 and *X*^2^ = 11.04, *p* = 0.012, respectively). Lysis at day 1 was similar as baseline lysis (*p* = 0.837), while titers at day 3 and day 7 were on average 20 and 13% higher than baseline titers, (*p* < 0.001 and *p* = 0.005, respectively; Figure 2A). Lysis at day 3 was on average 16% higher than day 1 (*p* = 0.008). In contrast to lysis, haptoglobin decreased after primary exposure, as concentrations at day 3 and day 7 were on average 14 and 11% lower, respectively, than baseline concentrations (*p* = 0.005 and *p* = 0.036; Figure 2C). Natural antibodies (agglutination) did not differ between baseline and the post-exposure days (*X*^2^ = 4.63, *p* = 0.201; Figure 2B).

All secondary-exposed laboratory mallards (*N* = 9) excreted virus upon primary and secondary LPAIV H4N6 exposure, albeit not daily (Figure 1B). At day 1 (1/3), day 3 (3/3), and day 7 (0/7) post-secondary exposure birds were excreting virus. Similar as with the primary-exposed group, blood samples were collected from three individuals per time point, i.e., day 1, 3, and 7 post-secondary exposure. All secondary-exposed laboratory mallards were positive for AIV-specific antibodies at secondary-baseline (i.e., day -1, 9/9), day 1 (3/3), day 3 (3/3), and day 7 (3/3) post-secondary exposure. Of the immune parameters, only complement activity (lysis) differed between the post-exposure days (*X*^2^ = 6.08, *p* = 0.048). Lysis on day 3 was on average 13% higher than at day 7 (*p* = 0.036; Figure 2D). Lysis at day 1, 3, and 7 upon secondary exposure did not differ from secondary-baseline (all *p* > 0.132). Natural antibodies (agglutination) and the acute phase protein haptoglobin did not differ between secondary-baseline and the post-exposure days (*X*^2^ = 4.04, *p* = 0.257 and *X*^2^ = 4.47, *p* = 0.215, respectively; Figures 2E,F).

### Natural Infection Study

Sentinel mallards (*N* = 9) were on average infected three times with LPAIV (viral prevalence of 4%, 29 out of 709 samples) during the autumn LPAIV infection peak. Blood sampling showed that all sentinels (9/9) were positive for AIV-specific antibodies in weekly collected samples throughout this period. For seven sentinels, we were able to estimate the day of virus exposure and link complement activity (lysis), natural antibodies (agglutination), and the acute phase protein haptoglobin to days 1–2, 3–4, and 6–8 post-LPAIV exposure. Lysis, agglutination, and haptoglobin upon natural exposure did not differ from baseline (based on sera collected when individuals tested negative for LPAIV for at least 20 days), nor was there any difference in values between days 1–2, 3–4, and 6–8 post-exposure (*X*^2^ = 0.59, *p* = 0.899, *X*^2^ = 5.76, *p* = 0.124 and *X*^2^ = 2.25, *p* = 0.522, respectively; Figures 2G–I).

Of all free-living mallards captured in the funnel live-trap daily during autumn, 25% (74 out of 298 samples) were infected with LPAIV and 77% (92 out of 120 samples) had AIV-specific antibodies. We could estimate the day of virus exposure and link the innate immune response to day 1, 2, and 3–4 post-LPAIV exposure for 23 of 60 individuals. Of these 23 birds, 69% (18 out of 26 samples) were positive for AIV-specific antibodies. Complement activity (lysis), natural antibodies (agglutination), and the acute phase protein haptoglobin did not vary between the post-exposure days (*F*_2,19_ = 0.94, *p* = 0.408, *F*_2,19_ = 0.43, *p* = 0.660, and *F*_2,18_ = 1.16, *p* = 0.337, respectively; Figures 2J–L).

### Comparing Experimental and Natural Infection Study

**Figure 3:**
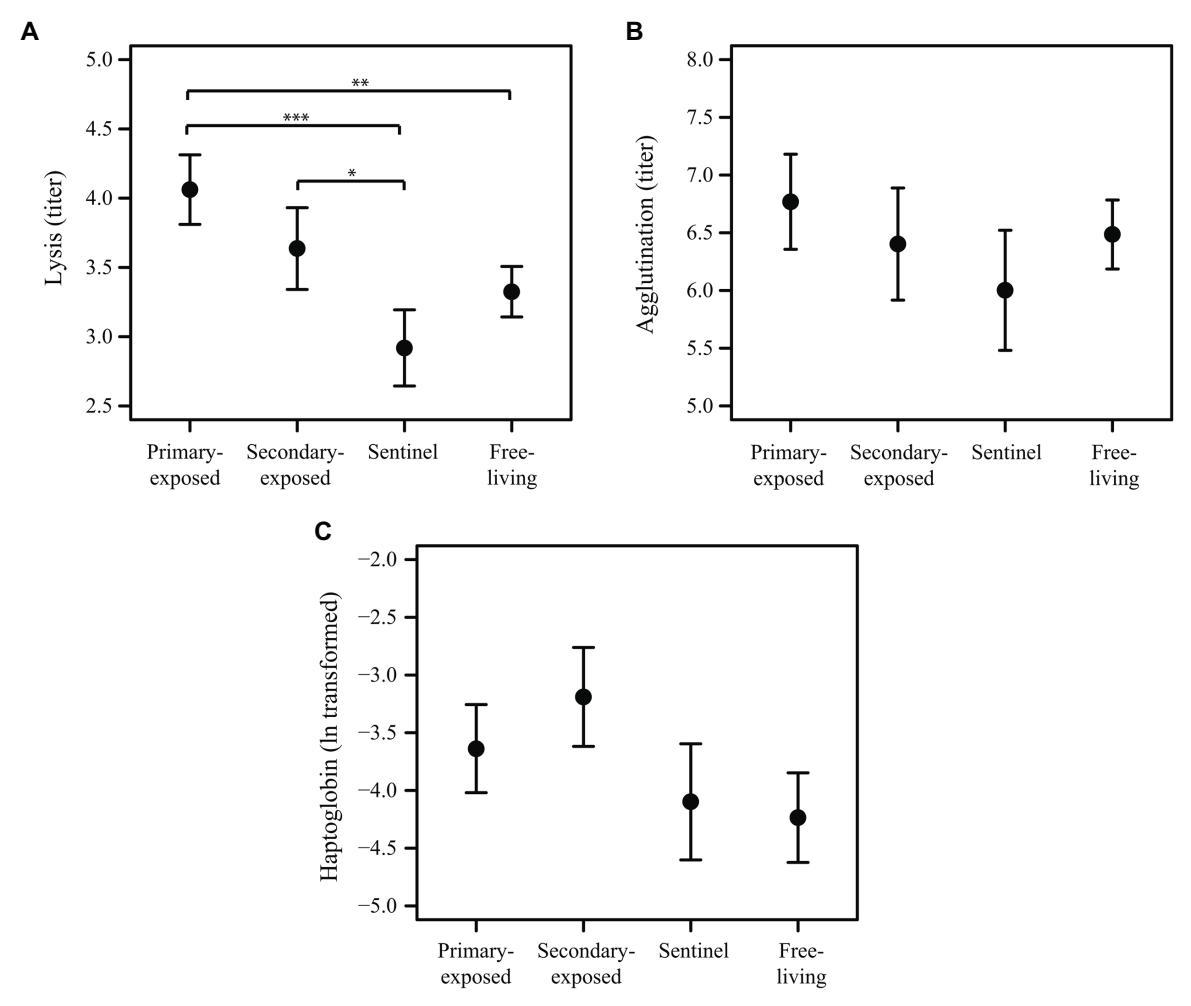
Innate humoral immune response in the first 3 days after LPAIV exposure (i.e., days 1–3 post-exposure merged) for primary-exposed laboratory (*N* = 6), secondary-exposed laboratory (*N* = 5), sentinel (*N* = 6), and free-living mallards (*N* = 25). Mean (±SE) of **(A)** lysis, **(B)** agglutination, and **(C)** haptoglobin. Significant codes represent: ^***^ = *p* < 0.001; ^**^ = *p* < 0.01; ^*^ = *p* < 0.05.

Complement activity (lysis) in the first 3 days after LPAIV exposure (day 1, 2, and 3 post-exposure were combined for each group) differed among primary-exposed laboratory, secondary-exposed laboratory, sentinel and free-living mallards (*X*^2^ = 17.49, *p* = 0.001). Lysis in primary-exposed laboratory mallards was on average 22% higher than in free-living mallards (*p* = 0.002), and 39% higher than in sentinel mallards (*p* < 0.001; Figure 3A). Lysis in secondary-exposed laboratory mallards was on average 25% higher than in sentinel mallards (*p* = 0.032), albeit secondary-exposed laboratory mallards had higher baseline lysis (10%) than sentinels (*F*_2,21_ = 3.74, *p* = 0.041). Natural antibodies (agglutination) and the acute phase protein haptoglobin in the first 3 days after LPAIV exposure did not differ between the four mallard groups (*X*^2^ = 2.25, *p* = 0.522 and *X*^2^ = 6.99, *p* = 0.072, respectively; Figures 3B,C). Baseline agglutination and baseline haptoglobin were similar among the three groups (*F*_2,21_ = 1.02, *p* = 0.377 and *F*_2,20_ = 0.98, *p* = 0.391, respectively).

## Discussion

Our study provides a unique opportunity to explore the innate humoral immune system and its interaction with LPAIVs in wild-type domestic mallards (both in the laboratory and in a semi-natural setting) and free-living mallards. We show that in primary-exposed laboratory mallards, LPAIV H4N6 likely activated the complement system (measured as lysis) and the acute phase response (measured as haptoglobin), as lysis was higher and haptoglobin lower at day 3 and 7 post-exposure compared to primary-baseline. The increase in lysis suggests that complement activity contributes to LPAIV neutralization. Our results are in accordance with the study of O’Brien et al. (2011), in which laboratory mice exposed to pandemic 2009 LPAIV H1N1 activated their complement system at day 3 post-infection to aid in viral clearance and regulation of lung inflammation. Experimental setups mimicking bacterial infections have shown that the complement system is activated within 24 h after exposure. Depending on the species (and potentially type, dose, route, and/or time interval), complement activity can increase, like in skylarks (*Alauda arvensis*; Hegemann et al., 2013), or decrease, like in Svalbard ptarmigan (*Lagopus muta hyperborea*; Nord et al., 2020) and crucian carp (*Carassius carassius*; Vinterstare et al., 2019) upon an immune challenge. The timing of the lysis peak in our study might be associated with virus excretion (and potentially with host susceptibility), as all birds were excreting virus at day 3 and day 7 post-primary exposure. Lower haptoglobin concentrations after LPAIV exposure in primary-exposed laboratory mallards is in accordance to Dannemiller et al. (2017), who also reported lower haptoglobin concentrations after LPAIV H4N6 inoculation in comparison to baseline concentrations in wild-type domestic mallards. However, it should be noted that these birds had been naturally exposed to LPAIV H9 prior to the onset of their study (Dannemiller et al., 2017). A decrease in haptoglobin concentrations after an immune challenge is also reported in other avian species (Mazur-Gonkowska et al., 2004; Troisi et al., 2007; Hegemann et al., 2013), as well as a fish species (Vinterstare et al., 2019). In contrast to mallards, haptoglobin in chickens increased after being infected with LPAIV (Sylte and Suarez, 2012; Dadras et al., 2014). Besides the fact that these are different species, other, not mutually exclusive, explanations could be differences in the time curve of the immune response, or in the timing that post-infection samples were collected.

LPAIV H4N6 infection did not seem to stimulate natural antibody production (measured as agglutination) in primary-exposed laboratory mallards, as agglutination titers upon exposure were similar as baseline titers. Although we did not find changes in natural antibody titers after LPAIV exposure, these antibodies could well be involved in virus neutralization, together with complement, as shown in a study of naïve laboratory mice where natural IgM were binding to LPAIV H1N1 (Jayasekera et al., 2007). Assessing whether natural antibodies in naïve mallards are also involved in LPAIV neutralization, needs further investigation. Another explanation for not finding changes in natural antibody titers could be the small group size of primary-exposed laboratory mallards in our study, which may had been too low to detect differences in natural antibody titers upon LPAIV exposure in case these were small. On the other hand, natural antibody titers in avian blood do not always change upon an experimental challenge (Hegemann et al., 2013), or the time for IgM to be sufficiently high to be detected may be more than a week following antigen stimulation, as is shown in human blood (Flaherty, 2012). Natural antibodies can activate the complement system, and efficiently induce lysis of pathogens to which they bind (Baumgarth et al., 1999; Koppenheffer et al., 1999). Our findings may question the fact whether the complement system in wild-type domestic and free-living mallards is indeed activated by natural antibodies, or that the complement system is directly activated by LPAIV. Even though complement plays an important role in the innate immune system, the underlying molecular processes how natural antibodies activate complement-immune responses is largely unclear (Sharp et al., 2019).

Complement activity (lysis), the acute phase protein haptoglobin, and natural antibodies (agglutination) upon secondary exposure with LPAIV H4N6 in laboratory mallards did not differ significantly from secondary-baseline. Secondary-baseline for lysis, haptoglobin, and agglutination did not differ significantly from values at primary-baseline, therefore, we consider that these innate immune parameters initiated by primary exposure were back at baseline levels prior to secondary exposure. It has been suggested that due to structure and function of duck isotypes, virus-immunized Pekin ducks commonly lack secondary antibody effects, such as agglutination and complement fixation (Higgins and Warr, 1993). Duck IgY consists of a full-length IgY and a truncated IgY (or IgYΔFc), in which the truncated IgY does not participate in complement fixation, opsonization and is defective in antigen internalization that is required for generation of T cells (Magor, 2011). On the other hand, lysis at day 1, 3, and 7 post-secondary exposure showed a similar, statistically non-significant trend as day 1, 3, and 7 post-primary exposure, which could indicate a weak response to LPAIV exposure. Although differences in virus excretion between the two post-exposure groups could be due to variation in timing of infection caused by the contact exposure route, previous studies show that LPAIV load and the duration of excretion is indeed significantly reduced when domestic mallards are exposed a second time to the same or a different LPAIV subtype (Latorre-Margalef et al., 2017).

We found no differences in lysis, haptoglobin, and agglutination after natural LPAIV exposure in sentinel and free-living mallards, suggesting these LPAIV pre-exposed birds were not responding to LPAIV infection by activating their complement system, acute phase response, and natural antibody production during our study period. Previously, a study in free-living mallards found no differences in lysis, haptoglobin, and agglutination between LPAIV-infected and uninfected birds (van Dijk et al., 2015). However, van Dijk et al. (2015) suggested that the innate immune response might still be upregulated (i.e., increased lysis, decreased haptoglobin) due to prior LPAIV infections in birds that tested AIV-negative. This could be the case in free-living mallards in our study as LPAIV infection history of these birds was unknown. On the other hand, we cannot exclude that sentinels and free-living mallards were (simultaneously) infected with other pathogens (e.g., other viruses or bacteria) that might have stimulated their immune system. A previous study showed that free-living mallards captured at the same location (Ottenby, Sweden) in autumn were infected with corona and paramyxoviruses besides LPAIVs (Wille et al., 2015). The differences in viral prevalence between sentinels (4%) and free-living mallards (25%) during the autumn period, may suggest that sentinels were less susceptible to infection with the LPAIVs circulating around the duck trap than their free-living counterparts. This hypothesis is partly supported by the fact that all sentinels had AIV-specific antibodies during this period in contrast to only three-third of the population of free-living mallards. In addition, viral prevalence of sentinels is based on repeated sampling of nine individuals that were all seropositive, which might explain the low infection rate. In other words, lower infection rates and higher antibody prevalence in sentinel than in free-living mallards may be explained by new, relatively more influenza-naïve free-living mallards being added over time. In sentinels, we recorded large variation in lysis (and in haptoglobin and natural antibodies) both at baseline and after LPAIV exposure, which is likely due to individual variation in infection history. Using the same sentinel compartment in the funnel live-trap and sampling methods as in our study, Wille et al. (2013) showed large individual variation in exposure history to LPAIV subtypes in sentinel mallards in autumn. We were unable to verify their findings as subtype determination of LPAIV-positive sentinels was low (four out of 29 samples) in our study.

Information of host and virus ecology is critical to build accurate epidemiological models to investigate the complex infection dynamics of LPAIVs in free-living mallard populations (Lisovski et al., 2018), to identify mechanisms of virus transmission (Breban et al., 2009) and to specify strategies for prevention and control of infectious diseases to protect animal and human health (Heesterbeek et al., 2015). However, in order to build realistic and meaningful epidemiological models, it is necessary to understand the strengths and limitations of the model species. Abolins et al. (2017) compared the immune response of pathogen-free laboratory mice with that of wild mice (*M. musculus*), showing that their innate immune response is remarkably similar despite the presumed expectation that the immune system of wild mice should be more active and competent, and where there were differences, the response of wild mice were suppressed. Here, we show that lysis in the first 3 days after LPAIV exposure was higher in primary-exposed laboratory mallards than in LPAIV pre-exposed sentinel and free-living mallards. Variation in activation of the complement system is most likely caused by differences in infection history, as immunologically naïve laboratory mallards were exposed to LPAIV for the first time, while sentinels and free-living mallards were most likely pre-exposed multiple times, with different LPAIVs and infective doses, to various LPAIV subtypes prior to the study (Wille et al., 2013; van Dijk et al., 2014). The high levels of pathogen exposure may suppress the innate immune response as a homeostatic mechanism to prevent immune-mediated pathology (Abolins et al., 2017). Although we corrected for differences in AIV-specific antibody status between the three mallard groups by including antibody status as a covariate in our models, we did not include acquired humoral (B-cells) or cell-mediated (T-cells) immunity induced by LPAIV or other pathogens. Secondary-exposed laboratory mallards were only exposed twice to LPAIV, therefore their innate immune responses seem to reflect those of sentinel and free-living mallards better than the immune response of primary-exposed laboratory mallards. We suggest that the innate immune response values of laboratory mallards after secondary LPAIV exposure, instead of response values resulting from a single exposure, should be incorporated in epidemiological models, at least in models estimating LPAIV infection dynamics of non-juvenile bird populations.

The difference in complement activity in the first 3 days after LPAIV exposure between sentinel and secondary-exposed laboratory mallards was most likely caused by differences in baseline values of lysis. Variation in infection history could also explain why baseline levels of lysis were lower in sentinels than in secondary-exposed laboratory mallards. Although we applied a LPAIV-free period of at least 20 days to calculate baseline lysis in sentinels, we cannot exclude that sentinels were infected with other viruses in this period. In contrast, baseline lysis in secondary-exposed laboratory mallards may also be increased, because they were kept in captivity. A study of house sparrows (*Passer domesticus*) in captivity showed that bacterial killing ability of plasma, a measure of innate immune function, was greater than pre-captive levels and increased with time spend in captivity (Love et al., 2017). Variation in age between secondary-exposed laboratory mallards (3–5 months) and sentinels (~1.5 year) could be a third potential explanation for the difference in baseline lysis between the two groups. In passerines, lysis develops during early ontogeny, but decreases at low rate over its lifetime once birds reach adulthood (Vermeulen et al., 2017). Sentinels had already reached maturity (mallards reach maturity at 1 year of age; Cramp and Simmons, 1977), while laboratory mallards had just completed their growth period, i.e., when most development in innate immune function occurs (Klasing and Leshchinsky, 1999; Matson et al., 2005), therefore, we cannot exclude that variation in age may have contributed for the difference in baseline lysis between the two groups.

In conclusion, based on the three innate humoral immune parameters measured in this study, domestic mallards seem an appropriate model to investigate innate immunology of their free-living counterparts, albeit results presented here suggest that the innate immune response upon LPAIV exposure of secondary-exposed laboratory mallards is likely a better proxy for the innate immune response in free-living pre-exposed mallards. Our study showed a weak but significant variation in innate humoral immune response upon LPAIV exposure among laboratory, sentinel, and free-living mallards, suggesting a potential for data transfer between experimental and natural LPAIV infection studies. Insights in the underlying mechanisms of mallards to limit and respond to LPAIV in the critical first few days after exposure – in addition to our current knowledge on their acquired humoral immune response to this virus (Kida et al., 1980; Costa et al., 2010; Fereidouni et al., 2010; Latorre-Margalef et al., 2013; van Dijk et al., 2014) – will increase our understanding of mallards’ defense system to LPAIV. This information will further our understanding of how mallards perpetuate LPAIV in nature, and increase our knowledge of their immune systems coevolutionary arms race with this virus (van Dijk et al., 2015). Also, from a veterinary perspective this is vital information, as mallards are an important species for zoonotic transmission and spillover events to domestic animals (Wille et al., 2017). Despite the acknowledged differences between the mallard groups in LPAIV exposure (infection history, infective dose, LPAIVs, and route of infection), we believe, we have taken an important first step in assessing the use of domestic mallards as an innate immunological model for their free-living counterparts in LPAIV, whereby we build a foundation on which future research on the overall immune response of mallards (e.g., immune genes, cytokines, and acquired immunity) can be built.

## Data Availability Statement

The raw data supporting the conclusions of this article will be made available by the authors, without undue reservation, to any qualified researcher.

## Ethics Statement

The animal study was reviewed and approved by Uppsala Research Animal Ethics Board (5.8.18-07998/2017) and Linköping Regional Ethics Board (2017-1068).

## Author Contributions

JD and JW designed the natural infection study. JV and JJ designed the experimental infection study. JD collected the blood samples of the natural infection study. JV executed the experiment. JD, JV, AH, CT, and JO performed lab analyses. JD, JV, and AH explored the data. JD conducted the statistical analyses. JD, JV, and AH wrote the original drafts with reviewing and editing provided by CT, JO, JJ, and JW. All authors contributed to the article and approved the submitted version.

### Conflict of Interest

The authors declare that the research was conducted in the absence of any commercial or financial relationships that could be construed as a potential conflict of interest.
